# Supplementary material for: Barren plateaus in quantum neural network training landscapes
Source: Nat Commun. 2018 Nov 16;9:4812. doi: 10.1038/s41467-018-07090-4 (PMC6240101; doi:10.1038/s41467-018-07090-4)
Supplement: Supplementary file 1 — Supplementary Information [file 41467_2018_7090_MOESM1_ESM.pdf]

Supplementary Information

Barren plateaus in quantum neural network training landscapes

McClean et al.

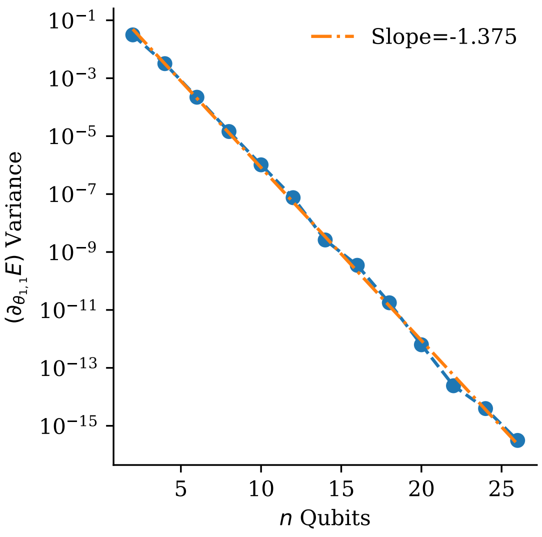

Supplementary Figure 1. Exponential decay of variance. The sample variance of the gradient of the energy for the first circuit component of the projection operator $H = |00...0\rangle \langle 00...0|$ plotted as a function of the number of qubits, n on a semi-log plot. As predicted, an exponential decay is observed as a function of the number of qubits for both the expected value and its spread. The slope indicates the rate of decay determined by the projection operator. The rate of decay of such projection operators is observed to be much higher than local observables, predicting challenges with respect to quantum state or circuit learning.

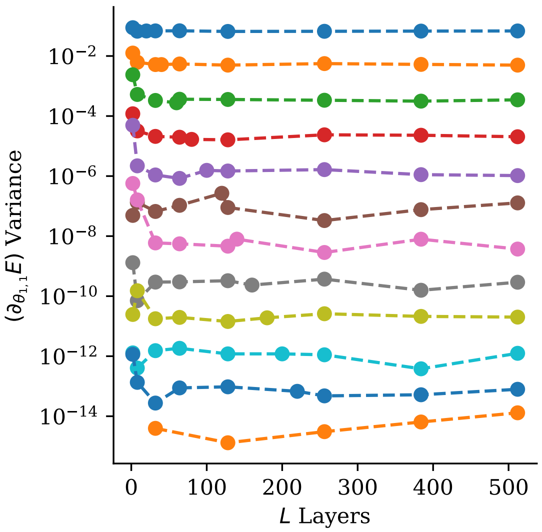

Supplementary Figure 2. Convergence to 2-design limit. The sample variance of the gradient of the energy for the first circuit component of the projection operator $H = |00...0\rangle \langle 00...0|$ plotted as a function of the number of layers, L, in the 1D quantum circuit. The different lines correspond to all even numbers of qubits between 2 and 24, with 2 qubits being the top line, and the rest being ordered by qubit number. This shows the convergence of the second moment as a function of the number of layers to a fixed value determined by the number of qubits. In contrast to local observables, the convergence of the variance is almost immediate, suggesting a substantially more challenging optimization problem.
